# The Bordeaux Conference

**Published:** 1924-07

**Authors:** 


### THE BORDEAUX CONFERENCE.

[By OUR SPECIAL CORRESPONDENT.]

The Annual Congress of the Royal Institute of Public Health was held at Bordeaux, from June 4 to June 9 inclusive, with Lord Burnham as President. The choice of Bordeaux as the seat of the Conference was fortunate; the fourth town of France in size, it is second only to Paris in political importance. The University is of considerable repute,—the number of undergraduates is just below 2,000, of whom about half are students of medicine. Doubtless owing to the city being a port, a medical school for the Naval Service is established there.

### Honours for English Doctors.

The afternoon before the Congress Sir Humphry Rolleston, President of the Royal College of Physicians, delivered a lecture on Endocrine Therapy before a large audience, comprising most of the members of the Faculty of Medicine. An inaugural service was held, on Wednesday morning, at the Cathedral of St. André. Lord Burnham placed a wreath on the War Monument in the Cathedral, and also on the monument placed in the Medical School in memory of Girondin medical men and students who lost their lives in the War. In the afternoon, at the inaugural meeting, the chief speakers were Lord Burnham and M. Philippart, the Maire of Bordeaux. The Maire, in an eloquent speech, deplored the present housing position, and pointed out that it could not fail to cause an increase of tuberculosis, alcoholism and anarchy. The Rector of the University, M. Dumas, informed the audience that Sir Humphrey Rolleston and Sir J. Bland Sutton had been awarded the honorary degree of Doctor of Medicine of the University.

### Treatment of the Tuberculous.

The meetings of the sections were well attended, especially those held in the two lecture theatres, where papers were read concerning municipal hygiene and Women and the Public Health. The latter section was presided over by Madame Wallerstein and Lady Bland Sutton; the French Government awarded the rosette of *Officier de l'Instruction Publique* to Lady Bland Sutton. In the section of Municipal Hygiene Prof. Achard dealt with vaccination of the civil population against typhoid. As long ago as 1912 and 1913 this measure had been adopted with success in the presence of epidemics in some twenty-four villages and small towns in France. Much time was given up to tuberculosis. The part played by dispensaries in the lessening of tuberculosis was rated highly; an example we might follow is the provision of facilities for disinfection and washing of the linen of open cases of tuberculosis. The French are evidently able to persuade tuberculous mothers to part with their newly born babies. Dr. Moussiet of Lyons pointed out the importance of health visitors making careful inquiries as to the presence of cases of open tuberculosis in the homes in which it was proposed to place out these babies. In industrial towns he thought that these infants were best placed in institutions. Lyons, carrying the separation further, had provided 1,000 beds in *preventoria* for such children when they reached the age of five years; the age of leaving is thirteen. An American visitor, Dr. Caroline Maule read one of the most valuable papers of the Congress. She gave an account of her experience in connection with the Schick test and preventive immunisation against diphtheria in New York (where she worked under Dr. W. H. Park) and at the Infants' Hospital, Westminster. Dr. Thomson (Deptford) and Dr. Hutt (Holborn) advocated the municipal adoption of this important measure at Infant Welfare Centres; the latter narrated the results attained at the Holborn Municipal Infant Welfare Centre during the last two years.

### Propaganda and Public Health.

In the discussion following the paper of Dr. Daley (Blackburn) on methods of public health propaganda, Sir Humphry Rolleston pointed out the importance of a well-balanced programme. Lectures given should deal not only with one disease but with several. Dr. Prosser White's paper dealt with the prevalence of bakers' itch. In the section on tropical medicine Professor G. Neveu Lemaire (Lyons) dealt with the possibility of destroying the molluscs which serve as the hosts of the larval stage of the parasite causing bilharziosis; Professor F. Mésnil (Paris) however thought this measure impracticable—certainly at present. In the section for Pathology, Bacteriology, and Biochemistry, Professor F. Bezançon (Paris) gave an account of his researches on the structure of the tubercle bacillus.

### Women and Public Health.

The most interesting papers in the section dealing with Women and the Public Health were those describing the work of the hospital almoner, by Mme. St. Luc, who gave an account of her training as a health visitor with nursing experience at the Florence Nightingale School, Bordeaux, and another by Mlle. Firmin, an *assistante sociale* at a children's hospital in Paris. Mlle. Firmin apparently acted as health visitor and social worker in connection with the patients seen at the hospital. Judging not only by the information derived from this Congress, but from several years' experience, it would be fair to say that the organisation of health work in France has by no means reached the heights attained in England. For example, Bordeaux, a town of some 250,000 inhabitants, has no *Directeur d'Hygiene*, although Dr. Llaguet, now the *Directeur d'Hygiene* of little Arcachon, will soon take up the duties.

### Exchange of Knowledge.

Visits were paid during the Congress to the Anti-Cancerous Centre of Bordeaux and the South-West, where inoperable cancer is treated by X-rays and radium by Professor Bergonié and his assistants; to the Military, Naval and Air Force Hygiene Exhibition; to Day Nurseries, the Florence Nightingale School, the health institutions of Arcachon, the saline baths at Biarritz, and the sulphurous baths at Luchon. The Congress certainly succeeded in bringing together French and British workers interested in public health and promoting the exchange of knowledge between the two countries.

